# The Assessment of Fear of COVID-19 among the Elderly Population: A Cross-Sectional Study

**DOI:** 10.3390/jcm10235537

**Published:** 2021-11-26

**Authors:** Siddarth Agrawal, Mateusz Dróżdż, Sebastian Makuch, Alicja Pietraszek, Małgorzata Sobieszczańska, Grzegorz Mazur

**Affiliations:** 1 Department and Clinic of Internal Medicine, Occupational Diseases, Hypertension and Clinical Oncology, Wroclaw Medical University, Borowska St. 213, 50-556 Wroclaw, Poland; pietraszek.alicja@gmail.com (A.P.); grzegorz.mazur@umed.wroc.pl (G.M.); 2 Laboratory of RNA Biochemistry, Institute of Chemistry and Biochemistry, Freie Universität Berlin, Takustraße 6, 14195 Berlin, Germany; m.drozdz@fu-berlin.de; 3 Department of Clinical and Experimental Pathology, Wroclaw Medical University, K. Marcinkowskiego St. 1, 50-368 Wroclaw, Poland; sebastian.mk21@gmail.com; 4 Department of Geriatrics, Wroclaw Medical University, Marii Skłodowskiej-Curie St. 66, 50-369 Wroclaw, Poland; malgorzata.sobieszczanska@umed.wroc.pl

**Keywords:** fear, COVID-19, older adults, health anxiety, media

## Abstract

The prevailing COVID-19 pandemic has dramatically affected the mental health and well-being of individuals. This cross-sectional study aimed to assess the perceived fear of COVID-19 among older adults in Poland and identify subpopulations with the highest risk of potential mental health disorders. The study was conducted in November–December 2020 on 500 people aged ≥60 years (mean M = 67.9, standard deviation SD = 4.2). In order to collect information on participants’ characteristics and COVID-19-related information, they were asked to complete a questionnaire based on recorded telephone calls. Perceived fear of COVID-19 was measured using Fear of COVID-19 Scale (FCV-19S), which ranges from 7 to 35. Multiple linear regression was performed to identify factors associated with the perceived fear of COVID-19. Our results showed that the highest level of fear of COVID-19 infection was observed among women (*p* = 0.025) and patients taking anticoagulants (*p* = 0.004). Moreover, older adults with higher anxiety levels were more likely to be fearful of COVID-19 (according to the GAS-10 scale; *p* < 0.001). These findings may help policy makers and healthcare workers to adapt and implement better mental health strategies to help the elderly fight fear and anxiety during the prevailing pandemic.

## 1. Introduction

It has been almost 2 years since we first grappled with the COVID-19 pandemic caused by the severe acute respiratory syndrome coronavirus 2 (SARS-CoV-2). Despite many attempts to reduce viral transmission, the development of vaccines, new diagnostic methods, antiviral drugs, and treatment strategies, the virus still continues to take its toll. To date, approximately 242 million people have been infected worldwide, while around 5 million of them died due to COVID-19 infection [1]. The clinical symptoms are cough, high fever, fatigue, and shortness of breath. The elderly, immunocompromised, and/or those with pre-existing chronic diseases are at the highest risk to develop severe respiratory problems, leading to multi-organ failure, pneumonia, and death.

The observed high morbidity and mortality of SARS-CoV-2 have triggered the fear of COVID-19 infection. As an example, in a survey of 44,000 participants conducted in Belgium in April 2020, the number of people who reported anxiety (20%) or depression (16%) had increased substantially compared to the survey conducted in 2018 [2].

Furthermore, changes in daily lives and behavior and the implementation of social restrictions also led older adults to have increased stress and anxiety levels affecting their mental health conditions [3]. Studies determined four domains of fear: (1) fear of oneself or their family members getting infected, (2) fear of having economic losses and being unemployed, (3) fear of avoidance behaviors toward gaining knowledge about the pandemic, or (4) fear of making decisions on showing or not showing actions like whether to visit other family members or not, whether to look for information on death rates or not, etc. [4,5]. All these doubts may be seen by changes in sleep and eating patterns; worsening of psychiatric conditions including manifestations of passivity, impotence, resignation, exclusion, and anger in older adults; and increased rates of addictions to alcohol, tobacco, drugs, etc. [6]. Another risk factor associated with mental health concerns of COVID-19 infections is a social disconnection between older adults and their families and friends. The elderly population requires special care when it comes to adapting to new ways of medical consultations via telemedicine, as well as maintaining relationships with friends and families by internet-based technologies, e.g., video conferences [7,8]. It was also observed that mental health problems in those concerned about COVID-19 infection are often neglected in favor of psychological consultations with patients with other chronic diseases [9]. Furthermore, due to implemented social restrictions, the only possible way to update the COVID-19-related information among older adults is through media (TV, radio, newspapers). However, current media is thought to be bombarded by misinformation and false reports about the COVID-19 infection and, in turn, may cause unfounded fears among many netizens [10,11]. As older adults tend to spend more time watching television than younger counterparts, this group is at higher risk of expressing fear and anxiety of COVID-19 infection.

Elderly patients and those with pre-existing chronic diseases are at the highest risk of COVID-19 morbidity and mortality, and, hence, the fear and anxiety levels are the highest. Our previous study determined how fear of COVID-19 infection influenced the professional, social, and recreational activities in the elderly population in Poland. For instance, we determined that 10% of participants (50/500; 10%) canceled planned hospitalization due to the fear of COVID-19 infection. It was observed mainly in patients suffering from chronic heart and lung diseases [12]. This behavior further increases the risk of death, especially among the elderly population with a history of diseases. In this study, we aimed to assess the fear of COVID-19 infection in the elderly population and identify subpopulations that require special care, for instance, through counselling and/or family support.

## 2. Materials and Methods

### 2.1. Study Design

The survey was conducted in November–December 2020 during the second wave of the COVID-19 pandemic in a group of 500 people, including 290 women (58%) and 210 men (42%), aged 60 years and more (mean M = 67.9, standard deviation SD = 4.2). Respondents were asked to complete a questionnaire based on recorded telephone calls. The response rate was 40%. A stratified sampling per the demographic structure of a voivodeship was used to obtain a representative sample of the elderly population. The proper size of the sample using the following formula:

$$\mathrm{Sample}\;\mathrm{size}=\frac{Z_{1-a/2}{}^{2}p\left(1-p\right)}{d^{2}}$$

where

$Z_{1-a/2}$ is the standard normal variate (at 5% type 1 error *p* < 0.05), -1.96;
*p* is the expected prevalence obtained from a pilot study, -0.4; and
*d* is the absolute precision-0.043.

Target quotas were set for age and gender strata in each geographical region. The interviewers were properly trained and prepared to ensure the quality and accuracy of the interview. A data collection supervisor supervised all interviews, and a study coordinator randomly evaluated the recordings of the dialogue. The transcripts were not returned to participants for comment and/or correction nor were repeat interviews carried out. The duration of the interview ranged from 15 to 20 min. Participants provided their consent at the beginning of the interview. No compensation was provided for participating in the study. More details regarding the study design are shown in a previously published article [12]. The study was approved by the Bioethics Committee of Wroclaw Medical University.

### 2.2. Explanatory Variables

In order to answer the question “Which subpopulation of elderly patients are at the highest risk of COVID-19-related fear and anxiety?”, the questionnaire was divided into three sections. At first, we generated sociodemographic data from all respondents, including (1) gender (male/female), (2) age (categorized as 60–64 years; 65–69 years; 70 years and more), (3) place of residence (village; town, less than 20,000 inhabitants; town, between 20,000 to 100,000 inhabitants; town, between 100,000 to 200,000 inhabitants; town, between 200,000 to 400,000 inhabitants; town, more than 400,000 inhabitants), (4) household size (living alone, living with a partner, living with a partner and children, living with children, living with family, other situation), (5) education (primary, vocational, secondary, higher), (6) BMI (kg/m^2^ calculated based on the given body weight and mass), and household income per person per month (in Polish currency (PLN): less than 500PLN, 501–1000PLN, 1001–2000PLN, 2001–3000PLN, more than 3000PLN, refusal to answer) (Table S1). The second part of the questionnaire assessed the medical data of all respondents, including chronic diseases (e.g., coronary heart disease, diabetes mellitus, asthma, COPD, heart failure, kidney failure, gastroesophageal reflux disease), the number of drugs currently taken (1 to 3, 4 to 6, 7 to 10, more than 10), and prescribed medications (such as cardiac drugs, antihypertensive drugs, diuretics, analgesics, digestive ailments drugs, anticoagulants, antidepressants, and nootropics) (Table S2). In the third section, we assessed the physical conditions of respondents by validated scales such as (1) Activities of Daily Living scale (ADL), (2) the Lawton Instrumental Activities of Daily Living scale (IADL), (3) Abbreviated Mental Test Score (AMTS), (4) geriatric depression scale (GDS-15), (5) Geriatric Anxiety Scale (GAS-10), (6) Lubben Social Network Scale (LSNS-6), (7) social loneliness scale (Gierveld Scale), and (8) Mini Nutritional Assessment (MNA) (Table S3).

### 2.3. Measures

To assess the fear of COVID-19 in the senior population, we used “Fear of COVID-19 Scale” (FCV-19S) [13]. Participants stated their position in a questionnaire using a five point scale, ranging from “1—strongly disagree”, “3—neither agree nor disagree” to “5—strongly agree”. Hence, the cumulative score ranged from 7 to 35, where the higher the score, the greater the fear of COVID-19. The answers to the Fear of COVID-19 Scale are presented in Table 1.

Participants stated their position in a questionnaire using a five-point scale (ranging from “1 = strongly disagree”, “3 = neither agree nor disagree”, and “5 = strongly agree”). Hence, the cumulative score ranged from 7 to 35, where the higher the scores, the greater the fear of COVID-19. The answers to the Fear of COVID-19 Scale are presented in Table 1.

### 2.4. Statistical Analysis

To investigate the associations between the COVID-19-related fears and sociodemographic factors, and different physical and mental health conditions among our study group, descriptive statistics were calculated for continuous quantitative variables, and the non-parametric significance tests (Mann–Whitney U test and Kruskal–Wallis H test) were applied for qualitative variables (nominal and ordinal). Adjusted beta-coefficient (β) and 95% confidence interval (95% CI) were reported for regression analysis. All analyses were performed using the statistical software package Statistica v.13.3 (TIBCO Software Inc. Palo Alto, CA, USA). The *p*-values presented in the tables take into account the Bonferroni sequential correction (Holm–Bonferroni Method). A *p*-value of <0.05 was considered to be statistically significant.

## 3. Results

### 3.1. Participants’ Characteristics

The cross-sectional analysis included 500 patients (290 females, 58%, and 210 males, 42%) aged 60 and more (mean M = 67.9 ± 4.2). Most of them lived in a town, between 20,000 to 100,000 inhabitants (136/500; 27.2%), and fewer in villages (110/500; 22.0%). Including housing situation, 202 respondents lived with a partner (202/500; 40.4%). In general, most of the respondents were relatively highly educated; only eight people had a primary education (8/500; 1.6%), while 105 reported vocational education (105/500; 21.0%). Based on the given measurements of body weight and height, we calculated the body-mass index (BMI) of all participants (mean M = 27.4 ± 4.6). According to the World Health Organization (WHO) report, this result shows respondents were slightly overweight [14]. Taking into account household income per person per month, five people earned less than 500PLN (5/500; 1.0%), 24 people earned between 501PLN to 1000PLN (24/500; 4.8%), 188 people earned between 1001 to 2000PLN (188/500; 37.6%), 158 people earned between 2001 to 3000PLN (158/500; 31.6%), and 110 respondents earned more than 3000PLN (110/500; 22.0%). Furthermore, 15 respondents refused to answer this question (15/500; 3.0%). This result should be considered with caution, as, due to the restrictions, many people lost their jobs or had lowered salaries. Detailed data on the general characteristics of the surveyed people showing their sociodemographic data are presented in Table S1.

Most of the respondents suffered from one or more chronic diseases such as coronary heart disease (63/500, 12.6%), diabetes mellitus (74/500, 14.8%), asthma (43/500, 8.6%), chronic obstructive pulmonary disease (COPD) (33/500, 6.6%), heart failure (71/500, 14.2%), kidney failure (20/500, 4.0%), and gastroesophageal reflux disease (68/500, 13.6%). All participants took at least one medication regularly. Most of them took one to three drugs (301/500; 60.2%), while eight people took more than 10 medications (8/500; 1.6%). The most commonly taken medications were antihypertensive drugs (255/500, 51.0%) and analgesics (230/500, 46.0%), followed by cardiac drugs (132/500; 26.4%) and digestive ailments’ drugs (131/500, 26.2%). There were 352 participants who declared the same GP doctor always prescribed all drugs (352/500; 70.4%). The remaining respondents reported different doctors have prescribed medications (148/500; 29.6%). Detailed data on the clinical characteristics of the surveyed people are presented in Table S2.

According to the ADL scale, most of the participants were fit people (493/500; 98.6%). However, according to the GDS-15 scale, a significant number of patients suffered from depression (176/500; 35.2%). They exhibited less social engagement (according to LSNS-6 scale; mean = 14.2 ± 5.9) and felt lonely (according to the Gierveld Scale, mean = 13.1 ± 1.8). Most of the participants had a proper nutritional status according to the MNA scale (418/500, 83.6%). Detailed data on the psychological characteristics of the surveyed people are presented in Table S3.

### 3.2. Fear of COVID-19 Infection

**Figure 1:**
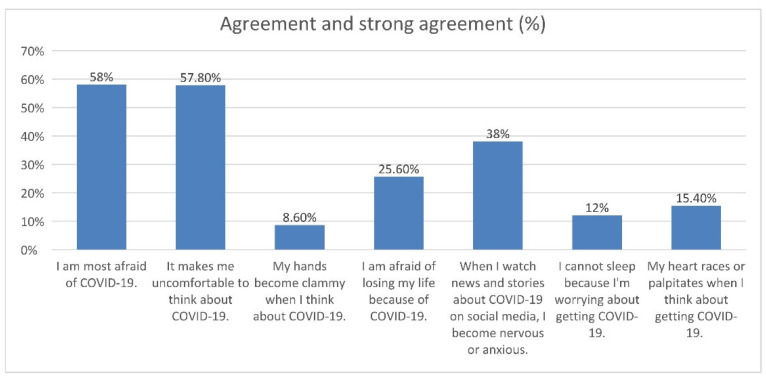
Participants agreement on seven items of The Fear of COVID-19 Scale (FCV-19S).

Many different factors contribute to the perception of fear and anxiety due to the prevailing COVID-19 pandemic, including sociodemographic factors, health conditions, and mental health. Based on the Fear of COVID-19 Scale (FCV-19S), we determined that 201 people were afraid of COVID-19 infection (201/500; 40.2%), and 89 participants were strongly afraid of COVID-19 infection (89/600; 17.8%). Eighteen people did not show any concerns about the pandemic (18/500; 3.6%); they did not care about the potential dangers of contact with other people. This result is in line with another question from FCV-19S, regarding feeling uncomfortable while thinking about COVID-19 infection. There were 220 participants who agreed (220/500; 44.0%) and 69 participants who strongly agreed with this statement (69/500; 13.8%). The fear of COVID-19 infection may be seen by the exhibition of different symptoms at different levels. Thus, other statements of FCV-19S included questions if patients’ hands become clammy when thinking about COVID-19 disease or if they had insomnia or rapid heartbeat because of worrying about COVID-19. However, according to our analysis, only 37 reported their hands become clammy when thinking about COVID-19 infection (27/500; 7.4%) and six people strongly agreed with this statement (6/500; 1.2%). Furthermore, 49 people reported suffering from insomnia, likely due to the threat of COVID-19 infection (49/500; 9.8%). For 14 people, it was obvious that insomnia was caused by the fear of getting sick (14/600; 2.8%). Fear of death caused by COVID-19 infection was observed in 94 respondents (94/500; 18.8%), and 34 people strongly agreed with this statement (34/500; 6.8%). The Fear of COVID-19 Scale also determined the impact of social media on the presence of anxiousness of COVID-19 infection. Watching news and stories regarding COVID-19 infection was the reason for the threat for 155 respondents (155/500; 31.0%). Participants’ reported agreement on the seven items of FCV-19 Scale is shown in Figure 1. It is worth noting many respondents reported “Neither agree nor disagree” for all statements and questions included in the questionnaire (Table 1).

Including all sociodemographic factors analyzed in this study, we determined the most critical predictor to exhibit fear and anxiety due to COVID-19 infection in the univariate analysis was the female gender (*p* = 0.025, Table 2). Women, regardless of age, were more often concerned about contracting COVID-19 infection than men.

Furthermore, the fear of COVID-19 infection increased in respondents with a history of coronary heart disease (*p* < 0.001), COPD (*p* = 0.007), and heart failure (*p* < 0.001) (Table 3).

Including clinical characteristics of all respondents, we also determined that the number of medicines taken affects the fear of COVID-19 infection levels (*p* = 0.002). The most crucial predictors to exhibit fear were found in people who take cardiac drugs (0 < 0.001), antihypertensive drugs (*p* = 0.011), analgesics (*p* = 0.001), digestive ailments drugs (*p* = 0.005), anticoagulants (*p* = 0.004), and antidepressants (*p* = 0.043) (Table 4).

Including the physical state of the respondents, the fear of COVID-19 infection was primarily associated with lowered ability to perform complex activities (according to the IADL scale; *p* = 0.013), reduced mental acuity (according to the AMTS scale; *p* = 0.013), depression (according to the GDS-15 scale; *p* < 0.001), higher anxiety levels (according to the GAS-10 scale; *p* < 0.001), social isolation (according to the LSNS-6 scale; *p* = 0.006), and loneliness (according to the Gierveld scale; *p* = 0.004). Furthermore, respondents with the danger of malnutrition and malnutrition were more concerned about contracting COVID-19 infection (according to the MNA scale; *p* = 0.017) (Table 5).

A multivariate regression analysis was performed to select independent predictors of a high expected fear of COVID-19. Its results are presented in Table 6. The most crucial predictors to exhibit higher fear of COVID-19 infection levels are (1) female gender (*p* = 0.007), (2) anticoagulants (*p* = 0.041), and (3) anxiety levels (according to the GAS-10 scale; *p* < 0.001) (Table 6).

## 4. Discussion

The rapid spread of the COVID-19 infection throughout the world has led to the increase of mental health crises, generated by the perception of stress, anxiety, depressive symptoms, insomnia, and anger. Older adults are at the highest risk of COVID-19 morbidity and mortality. The COVID-19 fatality rate for those over 80 years of age increases fivefold [15], and, hence, it is understandable they are at a higher risk of COVID-19-related fear and stress. The findings from our study showed a significant role of COVID-19 infection in perceiving fears among the older population in Poland, with a mean fear score of 19.3 ± 5.6 on a seven-item fear scale (fear score ranged between 7 to 35) (Table 4). Furthermore, we observed a great variety in participants’ agreement of the COVID-19 fear scale (Figure 1), which may result from differential emotional responses to the prevailing pandemic. This phenomenon is likely due to the lack of compliance in the mass media about SARS-CoV-2 epidemiology, routes of transmission, prevention, and/or lack of sufficient knowledge and awareness of individuals about this viral disease [16,17,18].

Our previous study determined that 10% of all surveyed Polish elderly population (50/500; 10%) canceled planned hospitalizations due to the fear of COVID-19 infection. Untreated for chronic diseases, COVID-19-infected patients are at an increased risk of death. Thus, despite the paradoxical sincere willingness to reduce the rate of SARS-CoV-2 transmission, such situations may adversely affect the clinical health of patients [12]. In this study, instead of highlighting the consequences of the fear during the COVID-19 pandemic (e.g., changes in behavior), we found subpopulations that are at the highest risk to exhibit health anxiety during the current pandemic.

At first, we found that women are more frequently associated with higher stress, anxiety, and depression due to potential COVID-19 infection (*p* = 0.025; Table 2, and *p* = 0.007, Table 6). This finding is consistent with other studies reporting the gender differences in behavior caused by the prevailing pandemic [19,20,21]. The explanation of the gender-based heterogeneity in contributing fear of COVID-19 was reported by Hosen et al. [22]. Based on the cross-sectional study, they found more irresponsible behaviors towards the COVID-19 pandemic in males, which significantly decreases their consciousness about the potential infection of the virus. In contrast, women were more inclined to adjust to government-imposed social restrictions, such as movement restrictions, covering the mouth and nose in public places, quarantining, or using disinfectants to reduce viral transmission. This behavior results from increased consciousness and, hence, potential fears of COVID-19 infection [22]. Furthermore, women are more susceptible to social isolation [23]. During the current pandemic, more women used psychological counseling than men, and these consultations focused mainly on emotional issues [24,25]. Women, especially elderly ones, as caretakers of families, are worried about themselves and their relatives, which intensifies the fear of COVID-19 infection.

Our study indicates that the fear of COVID-19 infection increases in people with pre-existing chronic diseases, such as coronary heart disease (*p* < 0.001, Table 3), COPD (*p* = 0.007, Table 3), and heart failure (*p* < 0.001, Table 3), which is consistent with other studies [26,27]. For instance, in a study aiming to assess the levels of fear of COVID-19 infection performed by Al-Rahimi et al., the significant predictors turned out to be the type of chronic disease including Crohn disease, hypertension, and cardiovascular diseases [28]. It is very likely that COVID-19 may affect the course of the pre-existing diseases and increase mortality because the overall stress caused by the viral infection may influence the cardiac muscle [29]. Furthermore, the study from the United States also reported that around one-third of infected patients with COVID-19 had at least one chronic disease; the most common were cardiovascular diseases, followed by chronic lung diseases and diabetes [30]. Thus, these results indicate the fear of COVID-19 infection in people, especially elderly ones, with cardiac and pulmonary problems is justifiable.

Furthermore, an important predictor of the fear of COVID-19 infection is also the number of prescribed medicines. The more drugs taken every day, the higher the levels of health anxiety caused by COVID-19 infection. This was observed mainly in people taking cardiac drugs (*p* < 0.001, Table 4), antihypertensive drugs (*p* = 0.011, Table 4), analgesics (*p* = 0.001, Table 4), digestive ailments’ drugs (*p* = 0.005, Table 4), anticoagulants (*p* = 0.004, Table 4), and antidepressants (*p* = 0.043, Table 4). It is worth noting that people taking anticoagulants were the most frequently concerned about contagion during the COVID-19 pandemic (*p* = 0.041, Table 6). There is mounting evidence that COVID-19 causes abnormalities in blood clotting in the veins and arteries, leading to life-threatening strokes, heart attacks, and pulmonary embolism [31]. Thus, in this case, fears of COVID-19 infection are also justifiable.

Preventive measures to reduce the spread of COVID-19 transmission (e.g., lockdowns, social distancing, mask wearing, etc.) have paradoxically caused a wide range of negative consequences, including social disconnection, mental health problems, and lifestyle changes [32], leading to increased fear levels of COVID-19 infection. The US Centers for Disease Control and Prevention (CDC) estimates that, as of June 2020, nearly one-third of US adults were suffering from anxiety or depression [33]. This result is consistent with our analysis. The fear of COVID-19 infection was increased in people with weakened mental capacity (according to AMTS scale, *p* = 0.013, Table 5) and those feeling depressed (according to the GDS-15 scale, *p* < 0.001, Table 5), lonely (according to the Gierveld Scale, *p* = 0.004, Table 5), and with high levels of anxiety (according to the GAS-10 scale, *p* < 0.001, Table 5) and social isolation (according to the LSNS-6 scale, *p* = 0.006, Table 5). It is worth noting that the highest fear of COVID-19 infection was reported in those exhibiting a high risk of anxiety (*p* < 0.001, Table 6). The current literature confirms this result. For instance, Mistry et al., conducted a cross-sectional study among 1032 older Bangladeshi adults aged ≥60 years. They determined that fear of COVID-19 infection was higher among those who felt socially isolated [18]. This study, together with our findings, suggests that we should pay more attention to the psychological support of the older community members during the pandemic.

The rapid spread of the COVID-19 disease leading to high daily rates of new cases and deaths together with the bombardment of information to which citizens are submitted through the media can influence the development of mood disorders. This affects mainly the elderly population, which tends to spend more time watching the media (radio, television, newspapers) than younger people. Moreover, the inability to visit loved ones and be visited by them due to social restrictions also increases fear and anxiety [20]. According to our study, from a total of 500 patients, 190 of them declared that watching and reading news about COVID-19 on social media made them feel nervous and scared (155/500, 31.0% agreed; and 35/500, 7.0% strongly agreed with this statement) (Figure 1, Table 2). The observed relationship between media exposure and the fear of the COVID-19 infection creates opportunities for policy makers and journalists to affect excessive worries. For instance, all information about SARS-CoV-2 epidemiology, prevention, and treatment should be provided unambiguously, without sensationalism and disturbing images. Furthermore, it is crucial to advise the elderly to restrict their exposure to media coverage of the COVID-19 crisis and avoid sensational media, which may increase stress and decrease the well-being of individuals [34,35,36].

As we are now after the third wave of the COVID-19 pandemic (late summer and autumn 2021) and including the fact that data were collected during the second wave (November–December 2020), the question appears if there are some changes in perceiving fears due to COVID-19 infection between these two different seasons. The first wave of the COVID-19 pandemic (spring 2020) raised the alarm in society, mainly because of the lack of knowledge about the pathogenicity and routes of transmission of SARS-CoV-2. The second wave identified the country differences in incidence, prevalence, and mortality rates of COVID-19. Although there was a significant impact of developed vaccinations, the third wave further exposed varying social and financial differences in different countries [37]. Several studies focused on the characteristics of effects of viral disease in different seasons [38,39,40,41], but no study highlighted changes in perceiving fears of COVID-19 infection during different waves. For instance, Iftimie et al. reported differences in age range and severity of the disease between two periods of COVID-19 infection (March–June 2020 and July–October 2020). Patients in the second wave were younger, and the duration of hospitalizations and case fatality rates were lower than those in the first wave. Furthermore, more children, pregnant and post-partum women, and people with renal and gastrointestinal symptoms were COVID-19 infected in the second wave than in the first wave of the COVID-19 pandemic [38]. It is worth noting that the analysis of the differential perception of fear of COVID-19 infection is quite challenging because different study groups referred to different periods as waves of the COVID-19 pandemic, depending mainly on the country where the study was performed.

To reduce the fear of COVID-19, it would also be beneficial to implement effective and informative campaigns about the disease, focused on its prevention. This solution could be crucial for the elderly population, which feels defenseless to face the problematic situation of the pandemic and is afraid to be COVID-19 infected. Furthermore, older adults should be provided with support plans with effective measures to improve their standard of living, eating habits, and living conditions [20]. This strategy may contribute to enhancing their ability to cope with the prevailing pandemic. It would be advisable for clinic authorities and health professionals to instantly design and implement measures to alleviate these effects, which harm the mental health of their patients. It is already determined that those who received COVID-19-related information from health workers had lower fear scores [18]. This result shows health workers are trusted among the older population and provide information in a sympathetic manner. Thus, the role of health workers in decreasing the fear of COVID-19 infection and enhancing the well-being among the elderly population is incontestable.

### Limitations

Our study has some limitations. At first, data were obtained by completing the questionnaire based on recorded telephone calls and the response rate was relatively low, 40%. The second limitation is the cross-sectional nature of the study based on self-reports. This may limit the generalizability of our results to a wider population and claims about the directionality of the results. Additionally, the authors could not assess if there were any differences between those who did and did not reply to the telephone survey as no information regarding nonrespondents was available. Furthermore, respondents recalled answers to questions. These answers may be subject to recall bias. This increases the risk of overreporting or underreporting the actual fear of COVID-19 infection.

## 5. Conclusions

To the best of our knowledge, this is the first nationwide study providing important information about fear due to COVID-19 infection among Polish older adults. This study is relevant for policy makers and healthcare workers to determine subpopulations with the highest risk to react fearfully toward the prevailing COVID-19 pandemic and for journalists to be aware of the potential impact of their work.

## Figures and Tables

**Table 1:** Assessment of fear of COVID-19 infection, based on FCV-19S.

Questionnaire Item, *n* (%)	Statistics
1. I am most afraid of COVID-19, Me (IQR)	4 (3–4)
Strongly disagree (1 point)	18 (3.6%)
Disagree (2 points)	55 (11.0%)
Neither agree nor disagree (3 points)	137 (27.4%)
Agree (4 points)	201 (40.2%)
Strongly agree (5 points)	89 (17.8%)
2. It makes me uncomfortable to think about COVID-19, Me (IQR)	4 (3–4)
Strongly disagree (1 point)	22 (4.4%)
Disagree (2 points)	77 (15.4%)
Neither agree nor disagree (3 points)	112 (22.4%)
Agree (4 points)	220 (44.0%)
Strongly agree (5 points)	69 (13.8%)
3. My hands become clammy when I think about COVID-19, Me (IQR)	2 (1–2)
Strongly disagree (1 point)	192 (38.4%)
Disagree (2 points)	193 (38.6%)
Neither agree nor disagree (3 points)	72 (14.4%)
Agree (4 points)	37 (7.4%)
Strongly agree (5 points)	6 (1.2%)
4. I am afraid of losing my life because of COVID-19, Me (IQR)	3 (2–4)
Strongly disagree (1 point)	73 (14.6%)
Disagree (2 points)	120 (24.0%)
Neither agree nor disagree (3 points)	179 (35.8%)
Agree (4 points)	94 (18.8%)
Strongly agree (5 points)	34 (6.8%)
5. When I watch news and stories about COVID-19 on social media, I become nervous or anxious, Me (IQR)	3 (2–4)
Strongly disagree (1 point)	44 (8.8%)
Disagree (2 points)	124 (24.8%)
Neither agree nor disagree (3 points)	142 (28.4%)
Agree (4 points)	155 (31.0%)
Strongly agree (5 points)	35 (7.0%)
6. I cannot sleep because I’m worrying about getting COVID-19, Me (IQR)	2 (1–3)
Strongly disagree (1 point)	135 (27.4%)
Disagree (2 points)	199 (39.8%)
Neither agree nor disagree (3 points)	101 (20.2%)
Agree (4 points)	49 (9.8%)
Strongly agree (5 points)	14 (2.8%)
7. My heart races or palpitates when I think about getting COVID-19, Me (IQR)	2 (1–3)
Strongly disagree (1 point)	132 (26.4%)
Disagree (2 points)	175 (35.0%)
Neither agree nor disagree (3 points)	116 (23.2%)
Agree (4 points)	63 (12.6%)
Strongly agree (5 points)	14 (2.8%)
The total assessment of fear of COVID-19 infection (total points):	
M ± SD	19.3 ± 5.6
Me (IQR)	19 (15–23)
Min–Max	7–35

**Table 2:** Assessment of fear of COVID-19 infection in groups differing in socio-demographic characteristics, Me (IQR) [in red: the most statistically significant (*p*-value < 0.05) predictors of exhibiting fear of COVID-19 infection in the elderly population].

Feature (Variable)	Statistics
Gender:	
Female (*n* = 290)	20 (16–23)
Male (*n* = 210)	19 (14–22)
U Mann–Whitney test:	*p* = 0.025
Age	
60–64 (*n* = 141)	19 (15–23)
65–69 (*n* = 128)	20 (16–23)
70 and more (*n* = 231)	19 (15–23)
Kruskal–Wallis test:	*p* = 0.832
Place of residence	*p* = 0.644
Housing situation	*p* = 0.597
Education	*p* = 0.397
Household income per person per month	*p* = 0.982

**Table 3:** Assessment of fear of COVID-19 infection in groups differing in the disease history, Me (IQR) [in red: the most statistically significant (*p*-value < 0.05) predictors of exhibiting fear of COVID-19 infection in the elderly population].

Feature (Variable)	Statistics
Coronary heart disease:	
Yes (*n* = 63)	22 (18–26)
No (*n* = 437)	19 (15–22)
U Mann–Whitney Test:	*p* < 0.001
Diabetes Mellitus:	
Yes (*n* = 74)	21 (16–25)
No (*n* = 426)	19 (15–23)
U Mann–Whitney Test:	*p* = 0.068
Asthma:	
Yes (*n* = 43)	20 (16–26)
No (*n* = 457)	19 (15–23)
U Mann–Whitney Test:	*p* = 0.245
COPD:	
Yes (*n* = 33)	22 (19–26)
No (*n* = 467)	19 (15–23)
U Mann–Whitney Test:	*p* = 0.007
Heart failure:	
Yes (*n* = 71)	22 (18–26)
No (*n* = 429)	19 (15–22)
U Mann–Whitney Test:	*p* < 0.001
Kidney failure:	
Tak (*n* = 20)	23 (18–27)
Nie (*n* = 480)	19 (15–23)
U Mann–Whitney Test:	*p* = 0.077

**Table 4:** Assessment of fear of COVID-19 infection in groups differing in taken medications, Me (IQR) [in red: the most statistically significant (*p*-value < 0.05) predictors of exhibiting fear of COVID-19 infection in the elderly population].

Feature (Variable)	Statistics
Number of drugs currently taken	
1 to 3 (*n* = 301)	19 (15–22)
4 to 6 (*n* = 151)	21 (16–25)
7 to 10 (*n* = 40)	20 (16–25)
More than 10 (*n* = 8)	25 (20–28)
U Mann–Whitney Test:	*p* = 0.002
Cardiac drugs	
Yes (*n* = 132)	21 (17–25)
No (*n* = 368)	19 (15–22)
U Mann–Whitney Test:	*p* < 0.001
Antihypertensive drugs	
Yes (*n* = 255)	20 (16–24)
No (*n* = 368)	19 (14-22)
U Mann–Whitney Test:	*p* = 0.011
Diuretics	
Yes (*n* = 78)	20 (17–25)
No (*n* = 422)	19 (15–23)
U Mann–Whitney Test:	*p* = 0.060
Analgesics	
Yes (*n* = 230)	20 (16-24)
No (*n* = 270)	18 (15-22)
U Mann–Whitney Test:	*p* = 0.001
For digestive ailments drugs	
Yes (*n* = 131)	20 (17–24)
No (*n* = 369)	19 (15–22)
U Mann–Whitney Test:	*p* = 0.005
Anticoagulants	
Yes (*n* = 87)	20 (17–25)
No (*n* = 413)	19 (15–23)
U Mann–Whitney Test:	*p* = 0.004
Antidepresants	
Yes (*n* = 78)	20 (17–24)
No (*n* = 422)	19 (15–23)
U Mann–Whitney Test:	*p* = 0.043
To improve memory drugs:	
Yes (*n* = 54)	19 (16–24)
No (*n* = 446)	19 (15–23)
U Mann–Whitney Test:	*p* = 0.786
All drugs are prescribed by the same doctor	
Yes (*n* = 352)	19 (15–23)
No (*n* = 148)	19 (15–23)
U Mann–Whitney Test:	*p* = 0.684
How many different doctors have prescribed your medications?	
2 (*n* = 82)	19 (14–23)
3 (*n* = 52)	20 (15–23)
4 and more (*n* = 14)	21 (17–25)
Kruskal–Wallis Test:	*p* = 0.455

**Table 5:** Assessment of fear of COVID-19 infection in groups differing in physical and mental health measurements, Me (IQR) [in red: the most statistically significant (*p*-value < 0.05) predictors of exhibiting fear of COVID-19 infection in the elderly population].

Feature (Variable)	Statistics
Activities of Daily Living (ADL)	
Fit people (*n* = 493)	19 (15–25)
Moderately disabled people (*n* = 6)	23 (18–26)
Disabled people (*n* = 1)	34
Kruskal–Wallis Test:	*p* = 0.150
The Lawton Instrumental Activities of Daily Living (IADL)	
Fit people, ≥24 pts. (*n* = 361)	19 (15–22)
Less fit people, <24 pts. (*n* = 139)	21 (16–24)
U Mann–Whitney Test:	*p* = 0.013
Abbreviated Mental Test Score (AMTS)	
Normal condition, 7–10 pts., (*n* = 491)	19 (15–23)
Moderate disorder, 4–6 pts., (*n* = 9)	22 (18–23)
U Mann–Whitney Test:	*p* = 0.013
Geriatric depression scale (GDS-15)	
Lack of depression, 0–5 pts., (*n* = 324)	18 (15–22)
Depression, 6–15 pts., (*n* = 176)	22 (17–25)
U Mann–Whitney Test:	*p* < 0.001
Geriatric Anxiety Scale (GAS-10)	
Lower anxiety level, 0–5 pts., (*n* = 210)	17 (14–21)
Higher anxiety level, 6–25 pts., (*n* = 290)	21 (17–25)
U Mann–Whitney Test:	*p* < 0.001
Lubben Social Network Scale (LSNS-6)	
Lower, 16–30 pts., (*n* = 209)	19 (14–22)
Higher, 0–15 pts., (*n* = 291)	20 (16–24)
U Mann–Whitney Test:	*p* = 0.006
Social loneliness scale (Gierveld Scale)	
Lower, 14–18 pts., (*n* = 209)	18 (14–22)
Higher, 6–13 pts., (*n* = 291)	20 (16–23)
U Mann–Whitney Test:	*p* = 0.004
Mini Nutritional Assessment (MNA)	
Proper nutritional status, 12–14 pts., (*n* = 418)	19 (15–22)
The danger of malnutrition, 8–11 pts., (*n* = 78)	21 (17–27)
Malnutrition, 0–7 pts., (*n* = 4)	21 (16–24)
Kruskal–Wallis Test:	*p* = 0.017

**Table 6:** Values of regression coefficients for the assessment of fear of COVID-19 infection with predictors significant in the univariate analysis [in red: the most statistically significant (*p*-value < 0.05) predictors of exhibiting fear of COVID-19 infection in the elderly population].

Predictors of Fear of COVID-19 Infection	b	*p*	Beta	*p*
Female gender	1.24	0.015	0.124	0.007
Coronary heart disease	2.52	0.001	-	>0.05
COPD	2.54	0.012	-	>0.05
Heart failure	2.54	<0.001	-	>0.05
The number of currently taken medicines	1.27	<0.001	-	>0.05
Cardiac drugs	2.08	<0.001	-	>0.05
Antihypertensive drugs	1.33	0.008	-	>0.05
Analgesics	1.66	0.001	-	>0.05
Digestive ailments’ drugs	1.64	0.004	-	>0.05
Anticoagulants	2.02	0.002	0.095	0.041
Antidepressants	1.23	0.078	-	>0.05
The Lawton Instrumental Activities of Daily Living (IADL)	−0.263	0.015	-	>0.05
Abbreviated Mental Test Score (AMTS)	−0.110	0.678	-	>0.05
Geriatric depression scale (GDS-15)	0.444	<0.001	-	>0.05
Geriatric Anxiety Scale (GAS-10)	0.473	<0.001	0.359	<0.001
Lubben Social Network Scale (LSNS-6)	−0.127	0.003	-	>0.05
Social loneliness scale (Gierveld Scale)	−0.481	<0.001	-	>0.05
Mini Nutritional Assessment (MNA)	−0.680	<0.001	-	>0.05

b, linear regression coefficient; β, standardized multiple regression coefficients.

## Data Availability

The authors confirm that the data supporting the findings of this study are available within the article.

## Supplementary Materials

The following are available online at https://www.mdpi.com/2077-0383/10/23/5537/s1. Table S1: Sociodemographic data of all surveyed respondents. Table S2: Clinical characteristics of the surveyed respondents. Table S3: Physical characteristics of the surveyed respondents.

## References

[1] Johns Hopkins Coronavirus Resource Center COVID-19 Map. https://coronavirus.jhu.edu/map.html.

[2] Berete F., Braekman E., Bruggeman H., Charafeddine R., Demarest S., Drieskens S., Gisle L., Hermans L., Leclercq V., Van der Heyden J. (2021). Zesde COVID-19-Gezondheidsenquête Eerste Resultaten.

[3] Quadros S., Garg S., Ranjan R., Vijayasarathi G., Mamun M.A. (2021). Fear of COVID-19 Infection Across Different Cohorts: A Scoping Review. Front. Psychiatry.

[4] Schimmenti A., Billieux J., Starcevic V. (2020). The four horsemen of fear: An integrated model of understanding fear experiences during the COVID-19 pandemic. Clin. Neuropsychiatry.

[5] Taylor S., Landry C.A., Paluszek M.M., Fergus T.A., McKay D., Asmundson G.J.G. (2020). COVID stress syndrome: Concept, structure, and correlates. Depress. Anxiety.

[6] Mamun M.A., Sakib N., Gozal D., Bhuiyan A.I., Hossain S., Bodrud-Doza M., Al Mamun F., Hosen I., Safiq M.B., Abdullah A.H. (2021). The COVID-19 pandemic and serious psychological consequences in Bangladesh: A population-based nationwide study. J. Affect. Disord..

[7] Twenge J.M., Joiner T.E. (2020). Mental distress among U.S. adults during the COVID-19 pandemic. J. Clin. Psychol..

[8] Roy D., Tripathy S., Kar S.K., Sharma N., Verma S.K., Kaushal V. (2020). Study of knowledge, attitude, anxiety & perceived mental healthcare need in Indian population during COVID-19 pandemic. Asian J. Psychiatry.

[9] Vahia I.V., Jeste D.V., Reynolds C.F. (2020). Older Adults and the Mental Health Effects of COVID-19. JAMA.

[10] Van Bavel J.J., Baicker K., Boggio P.S., Capraro V., Cichocka A., Cikara M., Crockett M.J., Crum A.J., Douglas K.M., Druckman J.N. (2020). Using social and behavioural science to support COVID-19 pandemic response. Nat. Hum. Behav..

[11] Su Z., McDonnell D., Wen J., Kozak M., Abbas J., Šegalo S., Li X., Ahmad J., Cheshmehzangi A., Cai Y. (2021). Mental health consequences of COVID-19 media coverage: The need for effective crisis communication practices. Glob. Health.

[12] Agrawal S., Makuch S., Dróżdż M., Strzelec B., Sobieszczańska M., Mazur G. (2021). The impact of the COVID-19 emergency on life activities and delivery of healthcare services in the elderly population. J. Clin. Med..

[13] Ahorsu D.K., Lin C.Y., Imani V., Saffari M., Griffiths M.D., Pakpour A.H. (2020). The Fear of COVID-19 Scale: Development and Initial Validation. Int. J. Ment. Health Addict..

[14] Obesity and Overweight. https://www.who.int/news-room/fact-sheets/detail/obesity-and-overweight.

[15] World Health Organization COVID-19 Strategy Update. www.who.int/emergencies/en.

[16] Arora A., Jha A.K., Alat P., Das S.S. (2020). Understanding coronaphobia. Asian J. Psychiatry.

[17] Freckelton I. (2020). COVID-19: Fear, quackery, false representations and the law. Int. J. Law Psychiatry.

[18] Mistry S.K., Ali A.R.M.M., Akther F., Yadav U.N., Harris M.F. (2021). Exploring fear of COVID-19 and its correlates among older adults in Bangladesh. Glob. Health.

[19] Alsharawy A., Spoon R., Smith A., Ball S. (2021). Gender Differences in Fear and Risk Perception During the COVID-19 Pandemic. Front. Psychol..

[20] Rodríguez-Hidalgo A.J., Pantaleón Y., Dios I., Falla D. (2020). Fear of COVID-19, Stress, and Anxiety in University Undergraduate Students: A Predictive Model for Depression. Front. Psychol..

[21] Huang Y., Zhao N. (2020). Generalized anxiety disorder, depressive symptoms and sleep quality during COVID-19 outbreak in China: A web-based cross-sectional survey. Psychiatry Res..

[22] Hosen I., Pakpour A.H., Sakib N., Hussain N., Al Mamun F., Mamun M.A. (2021). Knowledge and preventive behaviors regarding COVID-19 in Bangladesh: A nationwide distribution. PLoS ONE.

[23] Senst L., Baimoukhametova D., Sterley T.-L., Bains J.S. (2016). Sexually dimorphic neuronal responses to social isolation. eLife.

[24] Zhang J.X., Wang Y.N., Yang X.D., Chen Z.Y., Gao W.B. (2004). The dynamic analysis of public mental health during SARS epidemic period. Int. J. Psychol..

[25] Levkovich I., Shinan-Altman S. (2021). The impact of gender on emotional reactions, perceived susceptibility and perceived knowledge about COVID-19 among the Israeli public. Int. Health.

[26] Bakioğlu F., Korkmaz O., Ercan H. (2020). Fear of COVID-19 and Positivity: Mediating Role of Intolerance of Uncertainty, Depression, Anxiety, and Stress. Int. J. Ment. Health Addict..

[27] Moghanibashi-Mansourieh A. (2020). Assessing the anxiety level of Iranian general population during COVID-19 outbreak. Asian J. Psychiatry.

[28] Al-Rahimi J.S., Nass N.M., Hassoubah S.A., Wazqar D.Y., Alamoudi S.A. (2021). Levels and predictors of fear and health anxiety during the current outbreak of COVID-19 in immunocompromised and chronic disease patients in Saudi Arabia: A cross-sectional correlational study. PLoS ONE.

[29] Guo T., Fan Y., Chen M., Wu X., Zhang L., He T., Wang H., Wan J., Wang X., Lu Z. (2020). Cardiovascular Implications of Fatal Outcomes of Patients with Coronavirus Disease 2019 (COVID-19). JAMA Cardiol..

[30] Chow N., Fleming-Dutra K., Gierke R., Hall A., Hughes M., Pilishvili T., Ritchey M., Roguski K., Skoff T., Ussery E. (2020). Preliminary Estimates of the Prevalence of Selected Underlying Health Conditions Among Patients with Coronavirus Disease 2019—United States, 12 February–28 March 2020. MMWR Morb. Mortal. Wkly. Rep..

[31] Chandra A., Chakraborty U., Ghosh S., Dasgupta S. (2021). Anticoagulation in COVID-19: Current concepts and controversies. Postgrad. Med. J..

[32] Nazir M., Hussain I., Tian J., Akram S., Tshiaba S.M., Mushtaq S., Shad M.A. (2020). A multidimensional model of public health approaches against COVID-19. Int. J. Environ. Res. Public Health.

[33] Czeisler M.É., Lane R.I., Petrosky E., Wiley J.F., Christensen A., Njai R., Weaver M.D., Robbins R., Facer-Childs E.R., Barger L.K. (2020). Mental Health, Substance Use, and Suicidal Ideation During the COVID-19 Pandemic—United States, 24–30 June 2020. MMWR Morb. Mortal. Wkly. Rep..

[34] Garfin D.R., Silver R.C., Holman E.A. (2020). The novel coronavirus (COVID-2019) outbreak: Amplification of public health consequences by media exposure. Health Psychol..

[35] Asmundson G.J.G., Taylor S. (2020). How health anxiety influences responses to viral outbreaks like COVID-19: What all decision-makers, health authorities, and health care professionals need to know. J. Anxiety Disord..

[36] Mertens G., Gerritsen L., Duijndam S., Salemink E., Engelhard I.M. (2020). Fear of the coronavirus (COVID-19): Predictors in an online study conducted in March 2020. J. Anxiety Disord..

[37] Rahman M.A., Islam S.M.S., Tungpunkom P., Sultana F., Alif S.M., Banik B., Salehin M., Joseph B., Lam L., Watts M.C. (2021). COVID-19: Factors associated with psychological distress, fear, and coping strategies among community members across 17 countries. Glob. Health.

[38] Iftimie S., López-Azcona A.F., Vallverdú I., Hernández-Flix S., de Febrer G., Parra S., Hernández-Aguilera A., Riu F., Joven J., Andreychuk N. (2021). First and second waves of coronavirus disease-19: A comparative study in hospitalized patients in Reus, Spain. PLoS ONE.

[39] Pedro S.A., Ndjomatchoua F.T., Jentsch P., Tchuenche J.M., Anand M., Bauch C.T. (2020). Conditions for a Second Wave of COVID-19 Due to Interactions Between Disease Dynamics and Social Processes. Front. Phys..

[40] Kunno J., Supawattanabodee B., Sumanasrethakul C., Wiriyasivaj B., Kuratong S., Kaewchandee C. (2021). Comparison of Different Waves during the COVID-19 Pandemic: Retrospective Descriptive Study in Thailand. Adv. Prev. Med..

[41] Seong H., Hyun H.J., Yun J.G., Noh J.Y., Cheong H.J., Kim W.J., Song J.Y. (2021). Comparison of the second and third waves of the COVID-19 pandemic in South Korea: Importance of early public health intervention. Int. J. Infect. Dis..

