# Correction: Agrawal et al. The Assessment of Fear of COVID-19 among the Elderly Population: A Cross-Sectional Study. *J. Clin. Med.* 2021, *10,* 5537

**DOI:** 10.3390/jcm11133593

**Published:** 2022-06-22

**Authors:** Siddarth Agrawal, Mateusz Dróżdż, Sebastian Makuch, Alicja Pietraszek, Małgorzata Sobieszczańska, Grzegorz Mazur

**Affiliations:** 1 Department and Clinic of Internal Medicine, Occupational Diseases, Hypertension and Clinical Oncology, Wroclaw Medical University, Borowska St. 213, 50-556 Wroclaw, Poland; pietraszek.alicja@gmail.com (A.P.); grzegorz.mazur@umed.wroc.pl (G.M.); 2 Laboratory of RNA Biochemistry, Institute of Chemistry and Biochemistry, Freie Universität Berlin, Takustraße 6, 14195 Berlin, Germany; m.drozdz@fu-berlin.de; 3 Department of Clinical and Experimental Pathology, Wroclaw Medical University, K. Marcinkowskiego St. 1, 50-368 Wroclaw, Poland; sebastian.mk21@gmail.com; 4 Department of Geriatrics, Wroclaw Medical University, Marii Skłodowskiej-Curie St. 66, 50-369 Wroclaw, Poland; malgorzata.sobieszczanska@umed.wroc.pl

## 1. Figure/Table Legend, Error in Figure/Table

In the original publication [1], there were mistakes in the legends for **Figure 1** and **Table 1**. The authors mistakenly translated the FCV-19S scale from Polish to English, confusing it with a scale of their authorship. The correct legends appear below. 

**Figure 1. Participants agreement on seven items of The Fear of COVID-19 Scale (FCV-19S**).

**Table 1. Assessment of fear of COVID-19 infection, based on FCV-19S.**

In the original publication, there was a mistake in **Figure 1** as published. The corrected **Figure 1** appears below. 

In the original publication, there was a mistake in **Table 1** as published. The corrected **Table 1** appears below. 

## 2. Missing Citation

In the original publication, [13] **Ahorsu, D.K.; Lin, C.Y.; Imani, V.; Saffari, M.; Griffiths, M.D.; Pakpour, A.H. The Fear of COVID-19 Scale: Development and Initial Validation. *Int. J. Ment. Health Addict.* 2020, 1–9, Epub ahead of print**, was not cited. The citation has now been inserted into **Section 2.3**, and should read:

“To assess the fear of COVID-19 in the senior population, we used “Fear of COVID-19 Scale” (FCV-19S) [13].”

## 3. Text Correction

There was an error in the original publication. The authors mistakenly translated the FCV-19S scale from Polish to English, confusing it with a scale of their authorship.

A correction has been made to **Abstract**:

**Line 7: **Perceived fear of COVID-19 was measured using Fear of COVID-19 Scale (FCV-19S), which ranges from 7 to 35.

A correction has been made to **Section 2.3**:

“To assess the fear of COVID-19 in the senior population, we used “Fear of COVID-19 Scale (FCV-19S) [13]. Participants stated their position in a questionnaire using a five point scale, ranging from “1—strongly disagree”, “3—neither agree nor disagree” to “5—strongly agree”. Hence, the cumulative score ranged from 7 to 35, where the higher the score, the greater the fear of COVID-19. The answers to the Fear of COVID-19 Scale are presented in Table 1.”

A correction has been made to **Section 3.2**:

“Many different factors contribute to the perception of fear and anxiety due to the prevailing COVID-19 pandemic, including sociodemographic factors, health conditions, and mental health. Based on the Fear of COVID-19 Scale (FCV-19S), we determined that 201 people were afraid of COVID-19 infection (201/500; 40.2%), and 89 participants were strongly afraid of COVID-19 infection (89/600; 17.8%). Eighteen people did not show any concerns about the pandemic (18/500; 3.6%); they did not care about the potential dangers of contact with other people. This result is in line with another question from FCV-19S, regarding feeling uncomfortable while thinking about COVID-19 infection. There were 220 participants who agreed (220/500; 44.0%) and 69 participants who strongly agreed with this statement (69/500; 13.8%). The fear of COVID-19 infection may be seen by the exhibition of different symptoms at different levels. Thus, other statements of FCV-19S included questions if patients’ hands become clammy when thinking about COVID-19 disease or if they had insomnia or rapid heartbeat because of worrying about COVID-19. However, according to our analysis, only 37 reported their hands become clammy when thinking about COVID-19 infection (27/500; 7.4%) and six people strongly agreed with this statement (6/500; 1.2%). Furthermore, 49 people reported suffering from insomnia, likely due to the threat of COVID-19 infection (49/500; 9.8%). For 14 people, it was obvious that insomnia was caused by the fear of getting sick (14/600; 2.8%). Fear of death caused by COVID-19 infection was observed in 94 respondents (94/500; 18.8%), and 34 people strongly agreed with this statement (34/500; 6.8%). The Fear of COVID-19 Scale also determined the impact of social media on the presence of anxiousness of COVID-19 infection. Watching news and stories regarding COVID-19 infection was the reason for the threat for 155 respondents (155/500; 31.0%). Participants’ reported agreement on the seven items of FCV-19 Scale is shown in Figure 1. It is worth noting many respondents reported “Neither agree nor disagree” for all statements and questions included in the questionnaire (Table 1).”

## 4. References

Reference No. 13 has been changed from [2] to [3].

The authors apologize for any inconvenience caused, and state that the scientific conclusions are unaffected. This correction was approved by the Academic Editor. The original publication has also been updated.

## Figures and Tables

**Figure 1:**
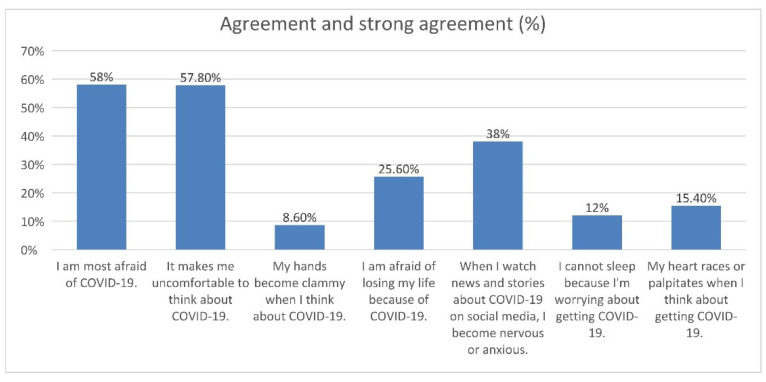
Participants agreement on seven items of The Fear of COVID-19 Scale (FCV-19S).

**Table 1:** Assessment of fear of COVID-19 infection, based on FCV-19S.

Questionnaire Item, *n* (%)	Statistics
1. I am most afraid of COVID-19, Me (IQR)	4 (3–4)
Strongly disagree (1 point)	18 (3.6%)
Disagree (2 points)	55 (11.0%)
Neither agree nor disagree (3 points)	137 (27.4%)
Agree (4 points)	201 (40.2%)
Strongly agree (5 points)	89 (17.8%)
2. It makes me uncomfortable to think about COVID-19, Me (IQR)	4 (3–4)
Strongly disagree (1 point)	22 (4.4%)
Disagree (2 points)	77 (15.4%)
Neither agree nor disagree (3 points)	112 (22.4%)
Agree (4 points)	220 (44.0%)
Strongly agree (5 points)	69 (13.8%)
3. My hands become clammy when I think about COVID-19, Me (IQR)	2 (1–2)
Strongly disagree (1 point)	192 (38.4%)
Disagree (2 points)	193 (38.6%)
Neither agree nor disagree (3 points)	72 (14.4%)
Agree (4 points)	37 (7.4%)
Strongly agree (5 points)	6 (1.2%)
4. I am afraid of losing my life because of COVID-19, Me (IQR)	3 (2–4)
Strongly disagree (1 point)	73 (14.6%)
Disagree (2 points)	120 (24.0%)
Neither agree nor disagree (3 points)	179 (35.8%)
Agree (4 points)	94 (18.8%)
Strongly agree (5 points)	34 (6.8%)
5. When I watch news and stories about COVID-19 on social media, I become nervous or anxious, Me (IQR)	3 (2–4)
Strongly disagree (1 point)	44 (8.8%)
Disagree (2 points)	124 (24.8%)
Neither agree nor disagree (3 points)	142 (28.4%)
Agree (4 points)	155 (31.0%)
Strongly agree (5 points)	35 (7.0%)
6. I cannot sleep because I’m worrying about getting COVID-19, Me (IQR)	2 (1–3)
Strongly disagree (1 point)	135 (27.4%)
Disagree (2 points)	199 (39.8%)
Neither agree nor disagree (3 points)	101 (20.2%)
Agree (4 points)	49 (9.8%)
Strongly agree (5 points)	14 (2.8%)
7. My heart races or palpitates when I think about getting COVID-19, Me (IQR)	2 (1–3)
Strongly disagree (1 point)	132 (26.4%)
Disagree (2 points)	175 (35.0%)
Neither agree nor disagree (3 points)	116 (23.2%)
Agree (4 points)	63 (12.6%)
Strongly agree (5 points)	14 (2.8%)
The total assessment of fear of COVID-19 infection (total points):	
M ± SD	19.3 ± 5.6
Me (IQR)	19 (15–23)
Min–Max	7–35
