# Supplementary material for: The Assessment of Fear of COVID-19 among the Elderly Population: A Cross-Sectional Study
Source: J Clin Med. 2021 Nov 26;10(23):5537. doi: 10.3390/jcm10235537 (PMC8658105; doi:10.3390/jcm10235537)
Supplement: Supplementary file 1 [file jcm-10-05537-s001.zip › jcm-1457548-supplementary.pdf]

Table S1. Sociodemographic data of all surveyed respondents

Feature (variable)	Statistical data
Gender	
Female	290 (58.0%)
Male	210 (42.0%)
Age	
60 – 64	141 (28.2%)
65 - 69	128 (25.6%)
70 and more	231 (46.2%)
Place of residence	
Village	110 (22.0%)
Town, less than 20,000 inhabitants	56 (11.2%)
Town, between 20,000 to 100,000 inhabitants	136 (27.2%)
Town, between 100,000 to 200,000 inhabitants	62 (12.4%)
Town, between 200,000 to 400,000 inhabitants	39 (7.8%)
Town, more than 400,000 inhabitants	97 (19.4%)
Housing situation	
Lives alone	108 (21.6%)
Lives with partner	202 (40.4%)
Lives with partner and children	117 (23.4%)
Lives alone with children	35 (7.0%)
Lives with family	29 (5.8%)
Other situation	9 (1.8%)
Education	
Primary	8 (1.6%)
Vocational	105 (21.0%)
Secondary	245 (49.0%)
Higher	142 (28.4%)
BMI (kg/m^2)	
M ± SD	27.4 ± 4.6
Me (IQR)	27 (24-30)
Min - Max	19 - 46
Household income per person per month	
< 500 PLN	5 (1.0%)
501 – 1000 PLN	24 (4.8%)
1001 – 2000 PLN	188 (37.6%)
2001 – 3000 PLN	158 (31.6%)
More than 3000 PLN	110 (22.0%)
Refusal to answer	15 (3.0%)

Table S2. Clinical characteristics of the surveyed respondents

Feature (variables)	Statistical data
Chronic diseases:	
Coronary Heart Disease	63 (12.6%)
Diabetes Mellitus	74 (14.8%)
Asthma	43 (8.6%)
COPD	33 (6.6%)
Heart Failure	71 (14.2%)
Kidney Failure	20 (4.0%)
Gastroesophageal Reflux Disease	68 (13.6%)
Number of drugs taken	
1 to 3	301 (60.2%)
4 to 6	151 (30.2%)
7 to 10	40 (8.0%)
More than 10	8 (1.6%)
Cardiac drugs	132 (26.4%)
Antihypertensive drugs	255 (51.0%)
Diuretics	78 (15.6%)
Analgesics	230 (46.0%)
Digestive ailments drugs	131 (26.2%)
Anticoagulants	87 (17.4%)
Antidepressants	78 (15.6%)
Nootropics	54 (10.8%)
All drugs are prescribed by the same doctor	352 (70.4%)
How many different doctors have prescribed your medications?	N = 148
2	82 (55.4%)
3	52 (35.1%)
4 and more	14 (9.4%)

Table S3. Physical characteristics of the surveyed respondents

Feature (variables)	Statistical data
Activities of Daily Living (ADL)	
M ± SD	5.9 ± 0.4
Me (IQR)	6 (6-6)
Min - Max	2 - 6
Fit people (5 – 6 pts.)	493 (98.6%)
Moderately disabled people (3 – 4 pts.)	6 (1.2%)
Disabled people (0 – 2 pts.)	1 (0.2%)
The Lawton Instrumental Activities of Daily Living (IADL)	
M ± SD	22.9 ± 2.3
Me (IQR)	24 (23-24)
Min - Max	11 - 24
Abbreviated Mental Test Score (AMTS)	
M ± SD	9.1 ± 1.0
Me (IQR)	9 (9-10)
Min - Max	5 - 10
Normal condition (7–10 pts.)	9.1 ± 1.0
Moderate disorder (4–6 pts.)	9 (9-10)
Geriatric depression scale (GDS-15)	
M ± SD	4.8 ± 4.0
Me (IQR)	4 (2-8)
Min - Max	0 - 15
Lack of depression (0–5 pts.)	324 (64.8%)
Depression (6–15 pts.)	176 (35.2%)
Gastric Anxiety Scale (GAS-10)	
M ± SD	7.2 ± 4.6
Me (IQR)	6 (4-10)
Min - Max	0 - 25
Lubben Social Network Scale (LSNS-6)	
M ± SD	14.2 ± 5.9
Me (IQR)	15 (10-18)
Min - Max	0 - 30
Social loneliness scale (Gierveld Scale)	
M ± SD	13.1 ± 1.8
Me (IQR)	13 (12-14)
Min - Max	6 - 18
Mini Nutritional Assessment (MNA)	
M ± SD	12.8 ± 1.5
Me (IQR)	13 (12-14)
Min - Max	6 - 14
Proper nutritional status (12–14 pts.)	418 (83.6%)
The danger of malnutrition (8–11 pts.)	78 (15.6%)
Malnutrition (0–7 pts.)	4 (0.8%)
